# Advantages of the Ilizarov external fixation in the management of intra-articular fractures of the distal tibia

**DOI:** 10.1186/1749-799X-4-35

**Published:** 2009-09-15

**Authors:** Elias S Vasiliadis, Theodoros B Grivas, Spyridon A Psarakis, Evangelos Papavasileiou, Angelos Kaspiris, Georgios Triantafyllopoulos

**Affiliations:** 1 Orthopaedic Department, "Thriasio" General Hospital, G. Gennimata Av. 19600, Magoula, Attica, Greece; 2 Orthopaedic Department, "Tzanio" General Hospital, Tzani & Afendouli str, 18536, Piraeus, Greece

## Abstract

**Background:**

Treatment of distal tibial intra-articular fractures is challenging due to the difficulties in achieving anatomical reduction of the articular surface and the instability which may occur due to ligamentous and soft tissue injury. The purpose of this study is to present an algorithm in the application of external fixation in the management of intra-articular fractures of the distal tibia either from axial compression or from torsional forces.

**Materials and methods:**

Thirty two patients with intra-articular fractures of the distal tibia have been studied. Based on the mechanism of injury they were divided into two groups. Group I includes 17 fractures due to axial compression and group II 15 fractures due to torsional force. An Ilizarov external fixation was used in 15 patients (11 of group I and 4 of group II). In 17 cases (6 of group I and 11 of group II) a unilateral hinged external fixator was used. In 7 out of 17 fractures of group I an additional fixation of the fibula was performed.

**Results:**

All fractures were healed. The mean time of removal of the external fixator was 11 weeks for group I and 10 weeks for group II. In group I, 5 patients had radiological osteoarthritic lesions (grade III and IV) but only 2 were symptomatic. Delayed union occurred in 3 patients of group I with fixed fibula. Other complications included one patient of group II with subluxation of the ankle joint after removal of the hinged external fixator, in 2 patients reduction found to be insufficient during the postoperative follow up and were revised and 6 patients had a residual pain. The range of ankle joint motion was larger in group II.

**Conclusion:**

Intra-articular fractures of the distal tibia due to axial compression are usually complicated with cartilaginous problems and are requiring anatomical reduction of the articular surface. Fractures due to torsional forces are complicated with ankle instability and reduction should be augmented with ligament repair, in order to restore normal movement of talus against the mortise. Both Ilizarov and hinged external fixators are unable to restore ligamentous stability. External fixation is recommended only for fractures of the ankle joint caused by axial compression because it is biomechanically superior and has a lower complication rate.

## Introduction

Treatment of intra-articular fractures of the distal tibia is challenging due to the difficulties they present in achieving anatomical reduction of the articular surface of the ankle joint and the instability that may occur due to ligamentous and soft tissue injury. Numerous methods of treatment for these fractures have been reported, including conservative treatment with cast, open reduction and internal fixation and the combination of different types of external fixators with or without internal fixation [1].

**Figure 1:**
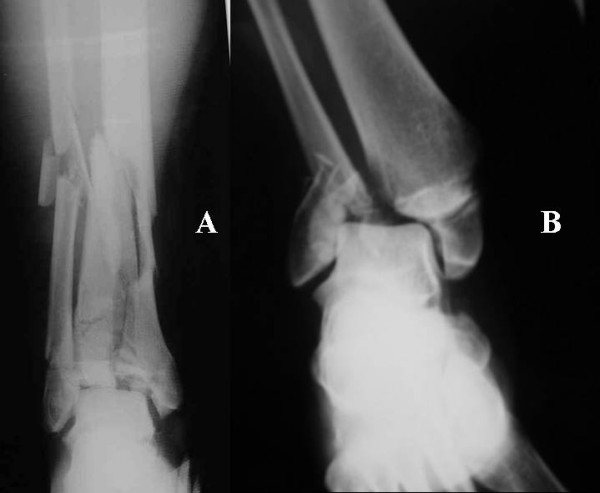
**(A). Typical distal tibia fracture due to axial compression (Pilon), (B) Intra-articular fracture of the ankle joint due to torsional force (bimaleollar)**.

Intra-articular fractures of the distal tibia are divided into two major groups. Those being caused by axial compression and those being as a result of torsional forces [2]. (Figure 1) The first group includes Pilon fractures, which are high energy fractures and are often complicated with severe soft tissue damage and postoperative articular surface defects due to the difficulties in anatomical restoration. The second group includes maleollar fractures, which are usually low energy fractures, are accompanied by smaller soft tissue injury and have as a major complication ankle instability due to ligament's tears.

Controversy exists in the literature concerning the way these fractures should be treated. The original classification of Pilon fractures by Ruedi and Allgower and the principles of treatment which they suggested, namely initial fibula fixation for length restoration, anatomical reduction of the articular surface, use of bone grafts in the metaphysis and finally internal fixation [3] was followed by high rate of complications, especially infection when the injury of soft tissues was severe [4,5]. This led many authors in treating these fractures in two steps, first by applying a temporary external fixation, followed by open reduction and internal fixation when the condition of soft tissues was improved [6].

Regarding torsional injuries of the ankle joint the classification by Lauge-Hansen correlates the type of fracture to the mechanism of injury and the anatomical defects and offers a treatment algorithm [7]. The Danis - Weber classification although is simpler its only contribution is in deciding to fix or not the tibiofibular syndesmosis.

Previously, the complex AO classification, which includes fractures resulting from both torsional and axial forces, led to confusion. For example, fractures which are classified as 'pronation - dorsiflexion' in Lauge - Hansen classification and are due to torsional forces, are classified as type B or C in AO classification. AO classification in combination with the treatment principles of Ruedi and Allgower it adopts [8], has led to incorrect treatment methods with increased rate of complications for the patients.

Recently the use of external fixation has radically changed the rate of complications of these fractures and improved their prognosis [9]. External fixators can be either unilateral or circular, they may span or not the ankle joint and may permit or not its motion.

The aim of the present study is not to introduce a new classification scheme, but to introduce an algorithm for the application of external fixation and to highlight the advantages of the Ilizarov device in the management of intra-articular fractures of the distal tibia.

## Materials and methods

This is a non randomized retrospective study of 32 patients with closed fractures of the distal tibia which were treated with external fixation. Inclusion criteria were age below 50 years, absence of concomitant fractures, treatment within 12 hours from admission and the use of external fixation. Polytrauma patients were excluded from the study.

Depending on the mechanism of injury, fractures were divided into two groups. Group I includes 17 fractures due to axial compression (5 fractures were type II and 12 fractures were type III according to Ruedi and Allgower's classification) in 13 male and 4 female patients with a mean age of 27,5 years (range 22 - 46) and mean follow up period of 21 months (range 14-28). Group II includes 15 fractures due to torsional forces (3 fractures due to supination/external rotation, 4 fractures due to pronation/external rotation and 8 fractures due to pronation/dorsiflexion according to Lauge - Hansen classification) in 10 male and 5 female patients with a mean age of 31,3 years (range 27-50) and mean follow up period of 19 months (range 12-28).

In 11 fractures of group I external fixation was applied as a neutralizing element combined with minor internal fixation for an anatomical articular surface reduction. Of these 11 fractures, 5 were type II and 6 were type III according to Ruedi and Allgower's classification. In all type II fractures and in one type III the neutralizing external fixator was a unilateral hinged external fixator, while in 5 fractures (type III) an Ilizarov device was used. In the remaining 6 fractures (all type III) an Ilizarov external fixation was applied as a major stabilization element after reduction due to ligamentotaxis. Fixation of the fibula was also performed in 6 out of 17 fractures in group I, where a unilateral external fixator was used.

**Figure 2:**
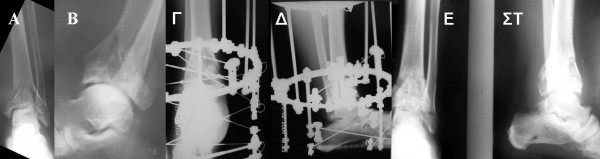
**Preoperative anteroposterior (A) and lateral (B) x ray of a distal tibial fracture due to axial compression (Pilon), treated with Ilizarov external fixation (Γ, Δ), with a good final result (E, ΣT)**.

**Figure 3:**
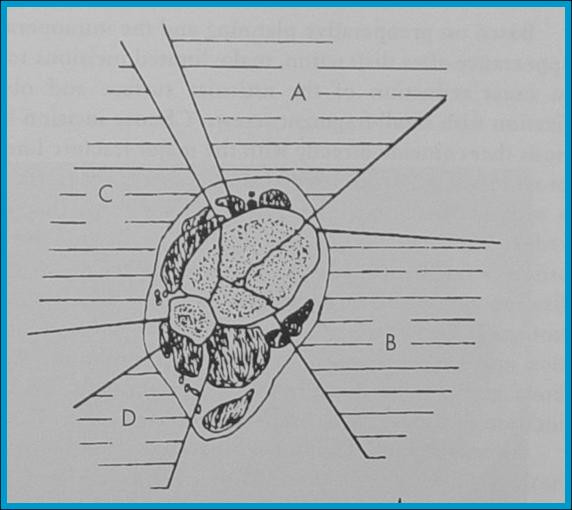
**Schematic representation showing the four main bone fragments in a distal tibial intra-articular fracture due to axial compression**. The anterior fragment on which the anterior articular capsule is attached **(A)**, the medial fragment which includes the medial maleollus **(B) **the lateral fragment, pulled by the tibiofibular syndesmosis **(Γ)**, the posterior fragment consisting of the posterior maleolus **(Δ)**.

**Figure 4:**
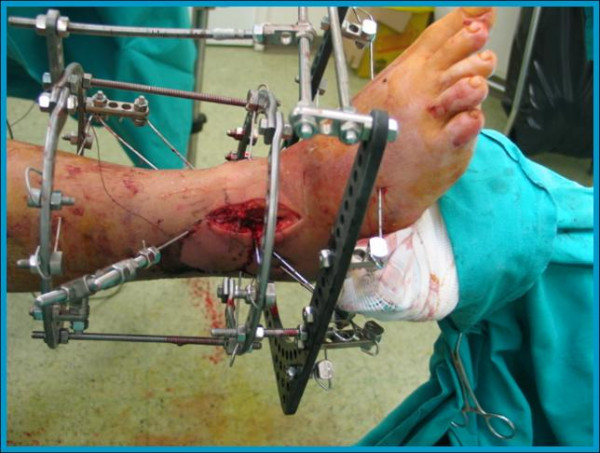
**Intraporative photograph showing the way the wires are applied and fixed to the rings of the Ilizarov device**. A small skin incision which was used for reduction of the articular surface is also visible.

The Ilizarov device consisted of 2 proximal rings placed at the distal half of the tibia and a foot plate. 1.8 mm olive wires have been used for the reduction and fixation of the major bone fragments and were properly connected to the rings. (Figure 2) Four major bone fragments were identified in this series of Pilon fractures. (Figure 3) The lateral fragment which consist an avulsion fracture of the tibiofibular syndesmosis, the medial fragment which includes the medial maleollus, the posterior fragment consisting of the posterior maleollus and the anterior fragment on which the anterior articular capsule inserts. With the Ilizarov device the fracture site is distracted and through ligamentotaxis the smaller bone fragments can be reduced and remain stable. Additionally, through the Ilizarov device the alignment of the limb is controlled, avoiding valgus or varus deformities. (Figure 4) Accuracy of reduction is controlled by image intensifier. No bone grafts were used. Early mobilization started 4-6 weeks postoperatively with the use of hinges at the ankle joint.

AO principles were followed in fractures which were treated with unilateral external fixators including fixation of the fibula, anatomic reduction of the articular surface, internal fixation of the fracture, occasionally use of bone grafts and finally stabilization with a unilateral external fixator. The proximal part of the devise was stabilized with the use of 3 half-pins into the tibial shaft and the distal part with 2 half-pins into the calcaneus and talus respectively, enabling at the same time motion of the ankle joint through a hinge which initially was locked.

In all the 15 patients of group II external fixation was applied as a major stabilizing element in unstable torsional injuries. Four were treated with an Ilizarov external fixator and 11 with a hinged unilateral external fixator.

In fractures were a unilateral device was used an open reduction and fixation of maleollar fractures was performed first. The main criterion for the application of external fixation was the clinical evaluation of the ankle joint stability intraoperatively. In all of these fractures external fixation found to be necessary in order to ensure joint stability through ligamentotaxis. No ligamentous repair was performed. The fixator which was used was the one described previously for patients of group I.

The selection of the Ilizarov devise for the treatment of torsional injuries of the ankle joint was based on bad soft tissue condition which did not allow open reduction (3 patients) or where x-rays were contraindicated (1 pregnant patient), where open reduction was performed through small incisions and no use of x-rays. Maleollar fractures were fixed with the use of olive wires properly adjusted to the Ilizarov frame, as it was described for patients of group I.

Patients were followed up clinically and radiographically. Accuracy of post operative reduction and ankle alignment were performed by plain x-rays. Postoperative evaluation included the presence of osteoarthritic lesions of the ankle joint, the residual ankle instability, range of motion, infection, time of union and time of removal of the device as well as the number of revision operations required.

## Results

All fractures were healed. The mean time for removal of the device was 11 weeks for group I (range 10-14) and 10 weeks for group II (range 9-11).

No patient had deep infection. Pin tract infection was the most common complication and was treated with frequent changes of the dressings and per os antibiotic administration.

**Figure 5:**
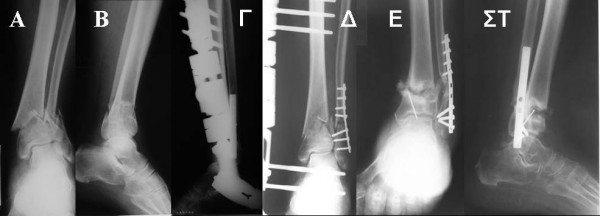
**Preoperative (A, B) and postoperative (Γ, Δ) x rays of a distal tibial fracture resulting from axial compression, that has been treated with fixation of the fibula according to the AO principles and complicated with delayed union (E, ΣT)**.

**Figure 6:**
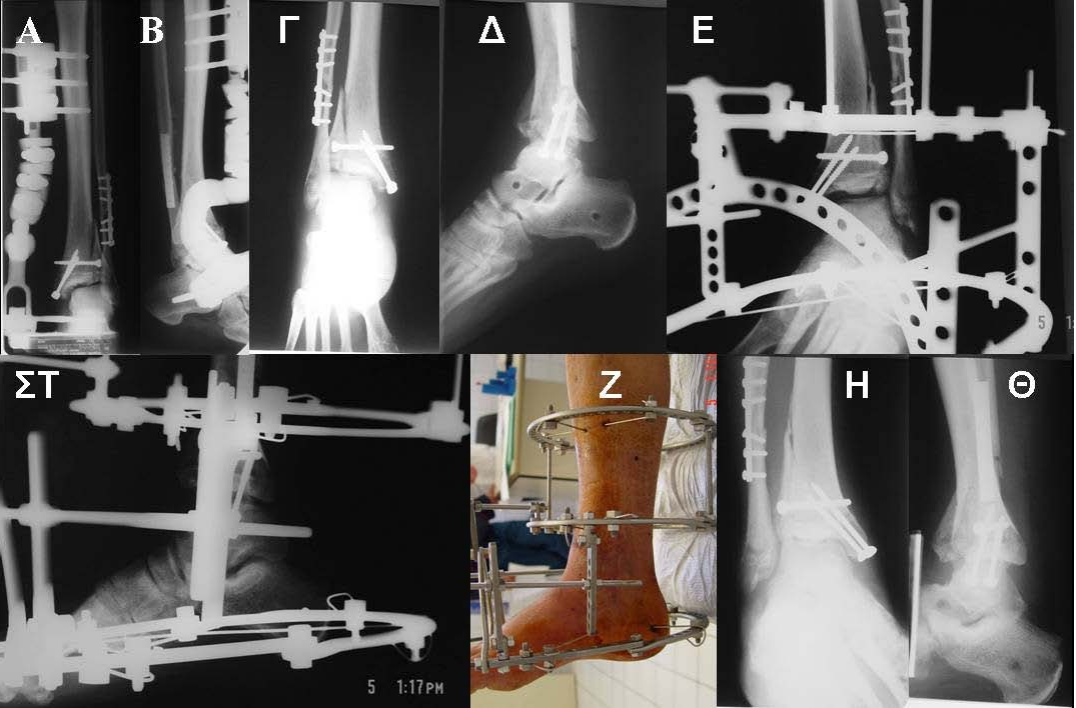
**Postoperative x rays (A, B) of a distal tibial fracture resulting from torional force, that has been treated with fixation of the fibula according to the AO principles**. Sublaxation of the ankle joint was revealed after removal of the unilateral external fixator **(Γ, Δ)**. It has been treated with aplication of an Ilizarov device **(E, ΣT) **for a gradual reduction of the subluxation with the proper placement of device's bars **(Z)**. Final x ray **(H, Θ) **showing the final result and the anatomical talus - tibia relation.

Five patients of group I were found with grade III and IV radiological osteoarthritic lesions of the ankle joint but only two of them were symptomatic and underwent ankle arthrodesis. In patients of group I, dorsiflexion of the ankle joint was restricted at an average of 20°. In 3 patients of group I who had their fibula fixed, a delayed union occurred, 3-5 months after removal of the external fixator. (Figure 5) One fracture from group II complicated with anterior subluxation after removal of the device and was re-operated because of unawareness of the mechanism of injury and underestimating the ligamentous instability of the ankle joint (Figure 6). In 2 patients of group II postoperative follow up revealed inadequate reduction and were re-operated, while in 6 patients residual pain was their major complaint. The range of motion was better in patients of group II.

## Discussion

Understanding the mechanism causing the distal tibia fracture is of major importance in order to choose the optimal method of treatment. The differences regarding the treatment principles between fractures caused by axial compression and those caused by torsional forces, render these two types of fractures totally different to each other, despite of the fact that they are sited at the same anatomic region.

The application of external fixation as a definite treatment for Pilon fractures has radically changed their prognosis [10-15]. By avoiding soft tissue detachment required for open reduction of the fracture, minimizes soft tissue injury, decreases infection rate [16] and permits early mobilization of the ankle joint through hinges in a stable mechanical environment [17].

The first step before the application of the external fixation is anatomical reduction of the articular surface. In order to achieve this, a small skin incision is required. The fragments are then fixed to their anatomical place by olive wires adjusted properly to the external fixator. The use of internal fixation is rarely required while the use of bone grafts is very limited.

Fixation of the fibula in fractures caused by axial compression which are treated by external fixation is not indicated. Anatomical reduction of the fibula does not allow fragment contact at the distal tibia metaphysis and has been associated with high incidence of delayed union or pseudarthrosis [18]. For open reduction and internal fixation of the fibula, one additional incision is required which may predispose to infection and at the same time reduction of the fibula itself may cause varus deformity. The stability of the ankle joint is not enhanced by fibula fixation because axial compression fractures are not accompanied by ligamentous damage [2]. If we reconsider that the major stabilizing element of the ankle joint is the deltoid ligament at the medial side [19], we can conclude that reduction and fixation of the fibula in such fractures has not a significant effect in the stability of the ankle joint.

The fractures of the distal tibia due to axial compression are often complicated by cartilage defects thus demanding an as good as possible anatomical reconstruction of the articular surface. Unfortunately, in many occasions besides of the large and relatively simple to fix fragments and the smaller ones which remain in place due to tension from ligamentotaxis, there are other smaller intra-articular bone fragments with no soft tissue attachments. These particles are responsible for the poor outcome regarding the articular surface and posttraumatic arthritis that may appear, because of their insufficient reduction or devascularization and high incidence of necrosis. However this outcome is not always accompanied by poor subjective clinical results [20].

Early mobilization of the ankle joint is another advantage of the Ilizarov device. In fractures caused by axial compression and no concomitant ligamentous instability, best results can be achieved, if mobilization is started 4-6 weeks postoperatively. Because the bone fragments are held in place by olive wires adjusted to the external fixation and there is not an additional independent internal fixation, intrafragmental microscopic motion is negligible and does not affect healing process. Although the 'in-frame' period is relatively high, especially for those fractures where external fixation applied as a neutralizing element, early mobilization through hinges, compensates the possible disadvantages of prolonged immobilization and enhances cartilage repair. The 4-6 week period until mobilization will start is considered to be sufficient to allow the development of a bone generating potential capable to lead to complete healing of the fracture.

In fractures caused by torsional forces the articular surface is usually easier to reconstruct by internal fixation. In this case, ankle instability, which is the major problem, induces postoperatively pain, while osteoarthitic lesions may appear later. Major concern in these fractures should be the restoration of the stability of the ankle joint by repair of the ligamentous elements. Essential goal is to restore all structures needed in order to achieve optimal talus movement in relation to the tibia. It is known that the body of the talus has the shape of a trapezoid and is wider anteriorly. When the foot dorsiflexes, the mortise is widened by a simultaneous posterolateral displacement and external rotation of the fibula. This synchronized motion performed by certain muscle activity, is controlled by mechanisms of proprioception through receptors of the ligaments and of the articular capsule and requires continuity of the ligaments and anatomical reduction of the articular surface [2].

All these parameters which were analyzed above are very difficult to be controlled by unilateral external fixators. When using olive wires of the Ilizarov device the bone fragments can securely be fixed. At the same time the talus, with an additional wire through its body can be centered in the mortise ensuring its symmetrical movement in relation to the tibia during full range motion of the ankle joint. This controlled mobilization can easily be done by using the correct hinges.

External fixation is contraindicated in most cases with fractures from tortional forces. Open reduction and internal fixation of these fractures combined with ligament repair is usually adequate. External fixation is recommended only for fractures of the ankle joint caused by axial compression, because only then it is biomechanically superior and results in a lower complication rate.

## Competing interests

The authors declare that they have no competing interests.

## Authors' contributions

EV conceived the idea of the presented study, performed part of the literature review and contributed in drafting of the manuscript and in the interpretation of data. TGB performed part of the literature review and contributed in the manuscript editing. SP, EP, AK and GT contributing in analyzing the data and in manuscript drafting. All authors have read and approved the final manuscript.
